# A New MAC Address Spoofing Detection Technique Based on Random Forests

**DOI:** 10.3390/s16030281

**Published:** 2016-02-24

**Authors:** Bandar Alotaibi, Khaled Elleithy

**Affiliations:** Computer Science and Engineering Department, University of Bridgeport, 126 Park Ave, Bridgeport, CT 06604, USA; elleithy@bridgeport.edu

**Keywords:** MAC address, spoofing, detection, random forests, wireless sensor networks, wireless local area networks

## Abstract

Media access control (MAC) addresses in wireless networks can be trivially spoofed using off-the-shelf devices. The aim of this research is to detect MAC address spoofing in wireless networks using a hard-to-spoof measurement that is correlated to the location of the wireless device, namely the received signal strength (RSS). We developed a passive solution that does not require modification for standards or protocols. The solution was tested in a live test-bed (*i.e.*, a wireless local area network with the aid of two air monitors acting as sensors) and achieved 99.77%, 93.16% and 88.38% accuracy when the attacker is 8–13 m, 4–8 m and less than 4 m away from the victim device, respectively. We implemented three previous methods on the same test-bed and found that our solution outperforms existing solutions. Our solution is based on an ensemble method known as random forests.

## 1. Introduction

The usage of wireless networks, such as wireless sensor networks (WSNs) and wireless local area networks (WLANs), have grown in recent years. WSN presents itself as a significant implementation for many applications due to its proficiency to monitor observations and report them to a central unit. Therefore, WSNs have been adopted by several applications, such as health monitoring and military surveillance. Additionally, WLANs have gained noticeable attention because of their ease of deployment and the availability of portable devices. Consequently, malicious attacks have increased enormously because of the shared medium that wireless networks use to serve wireless devices [1]. The media access control (MAC) address identifies wireless devices in wireless networks, yet it is susceptible to identity-based attacks. MAC address spoofing is an attack that changes the MAC address of a wireless device that exists in a specific wireless network using off-the-shelf equipment. MAC address spoofing is a serious threat to wireless networks. For instance, an attacker can spoof the MAC address of a productive access point (AP) in WLAN-infrastructure mode and replace or coexist with that AP to eavesdrop on the wireless traffic or act as a man-in-the-middle (this attack is known as the evil twin attack) [2,3,4,5,6]. In addition, the attacker can flood the network with numerous requests using random MAC addresses to exhaust the network resources. This attack is known as resource depletion [7,8,9].

These threats, along with other existing threats, necessitate the existence of MAC address spoofing detection to eliminate rogue devices. MAC address spoofing detection is very significant, because it is the first step to protect against rogue devices in wireless networks. Wireless networks (such as WSNs and WLANs) are integrated into a wide range of critical settings, including healthcare systems, such as mhealth applications, using machine-to-machine technology [10]. In addition, it is important to detect the presence of the rogue devices in wireless networks to protect smart grid systems, such as heating, ventilation and air conditioning (HVAC) systems [11]. The classical way to deal with spoofing is to employ authentication methods. Although authentication causes overhead and power consumption for wireless devices, it is even more costly to apply authentication to wireless devices that have limited resources. For instance, before authentication takes place (*i.e.*, before establishing the session keys to authenticate frames in a WLAN), the only identifier for a given wireless device is the MAC address. Thus, two devices in the same network that have the same MAC address are treated as legitimate clients, even though one of them has cloned the MAC address of the other.

In this article, we propose a solution that is based on the random forests ensemble method [12] and a hard-to-spoof metric, namely the received signal strength (RSS). Random forests-based approaches have been proposed in several applications and systems, including intrusion detection systems in the wired networks [13,14], spam detection [15] and phishing email detection [16]. However, random forests have not been used for similar issues as the one that we are solving in this article. Our problem depends entirely on the location of the legitimate and the attacker devices. The important feature that we utilize is the RSS that belongs to the physical layer. On the other hand, the wired intrusion detection systems (IDSs) utilize the upper layers, such as the application, transport and network layers; some important features are service type (*i.e.*, telnet, http or ftp), the presence of JavaScript and the number of links in the email. RSS measures the strength of the signal of the received packet at the receiver device. RSS can be affected by several factors, such as the transmission power of the sending device, the distance between the sender and receiver and some environmental elements, such as absorption effects and multi-path fading [17]. Normally, the wireless device does not change its transmission power, so the degradation of the signal from the same MAC address suggests the existence of MAC address spoofing [18]. We carried out an experiment in a “small office and home settings” live test-bed using WLAN devices to evaluate our proposed solution with the help of two air monitors acting as sensors. The sensors are capable of sniffing the wireless traffic passively and injecting traffic into the WLAN. We used the sensors to passively capture the wireless traffic and send it to the centralized utility for further analysis.

### 1.1. MAC Address Spoofing-Based Threats

An attacker can spoof the MAC address of a given legitimate user to hide his/her identity or to bypass the MAC address control list by masquerading as an authorized user. A more effective attack that the attacker can perform is to deny service on a given wireless network [19].

Deauthentication/disassociation: In the IEEE 802.11i standard, it is necessary to exchange the four-way handshake frames before an association takes place between a wireless device and the AP [20,21]. Once the station is associated with the AP, a hacker can disturb this association by sending a targeted deauthentication/disassociation frame to either disconnect the AP by spoofing the MAC address of the wireless user or disconnect the wireless user by spoofing the MAC address of the AP. A more harmful deauthentication/disassociation attack is to send frames to all of the wireless users using a broadcast address by spoofing the MAC address of the AP [22,23]. After sending the frame, the AP or the user who receives the frame is disconnected and has to repeat the entire authentication procedure in order to connect again. The attacker can also send spoofed deauthentication frames repeatedly to prevent the wireless user or the AP from maintaining the connection [24]. There are also other attacks, such as the power-saving attack that prevents the AP from queuing the upcoming frames for a given station by requesting these frames for a hacker instead of a legitimate station.

### 1.2. Attack Scenario

The attacker can spoof the MAC address of any device in the network, either as a wireless device or the AP. The attacker can change his/her transmission power, be mobile and be in close proximity to the legitimate device. The attacker could use a plug-and-play wireless card or a built-in wireless card. The attacker can inject packets into the network and can manipulate any packet field. Our aim is similar to [7,18], which is to profile the legitimate wireless device using RSS samples. We assume that a legitimate station is not mobile, which is true in some cases. For example, an AP in WLANs is in a fixed position. In the profiling period, we can actively send packets to a legitimate device to gather enough RSS samples to build up its normal profile. We also assume that there is no attacker during the profiling period.

### 1.3. Motivation

Many techniques have been proposed to detect MAC address spoofing, as it is a major threat to wireless networks. First, sequence number techniques [25,26] track the consecutive frames of the genuine wireless device. The sequence number increments by one every time the genuine device sends either data or management frame. Once the detection system finds an unexpected gap between two consecutive frames, the attacker is detected. Second, the operating system (OS) fingerprinting techniques [24] utilize the fact that some operating system characteristics could differentiate the attacker from the legitimate device when the spoofing occurs. Finally, RSS techniques [2,3,18,27,28] utilize the location of the legitimate device that should be different from the location of the attacker if they are not in the same location.

However, there are some limitations in the previous work. Sequence number approaches suffer from some drawbacks: one of the main types of MAC layer frames does not have sequence numbers, which is control frames. Thus, spoofing of control frames is possible. Furthermore, some of the tools used by the hackers provide the capability of eavesdropping and injecting frames that have sequence numbers similar to the frames of the legitimate device. OS fingerprinting techniques have some weaknesses, as well. The first weakness is that only the frame type that can be detected by the network layer’s OS fingerprinting is the data frame. The second weakness is that some of the techniques assume that the attacker spoofs the MAC address using Linux-based operating system tools. This assumption could cause some attackers to bypass the intrusion detection system. The attackers can use a capability that the Windows operating system provides to change the MAC address of a given user. Finally, vendor information, capability information and other similar fingerprinting techniques can be easily spoofed using off-the-shelf devices.

RSS approaches also have some limitations. Some researchers have reported that RSS samples from a given sender follow a Gaussian distribution, whilst other researchers revealed that the distribution is not Gaussian [29,30] or that it is not rare to notice non-Gaussian distributions of the samples [18]. As [18] reported, we found that it is not rare to find many peaks in the collected RSS samples. This suggests that the detection techniques [2,3,18,27,28] (based on clustering algorithms) that are closely related to our proposal are not the optimal solutions because these solutions assume that the samples are always Gaussian. Therefore, their solutions generate false alerts or miss some intrusions if the data are not Gaussian distributed. In addition, when the attacker and the victim devices are close to each other, the means/medians of both devices are close to each other, so distinguishing the two devices becomes hard. Furthermore, we discovered that in multiple cases, the distribution of the data from a single device constructs two clusters, so it is hard for the clustering algorithm-based approaches to perform well in these situations. Motivated by these concerns, we utilized a machine learning algorithm that can deal with both data that are Gaussian distributed and, more importantly, data that are not actually Gaussian distributed. Thus, in this article, we proposed a detection method based on random forests, because it can determine the dataset shape in order to obtain better results and the hard-to-spoof measurement (*i.e.*, the RSS).

This paper’s contributions can be summarized as follows:

1. We develop a new passive technique to detect MAC address spoofing based on the random forests ensemble method.
2. We compare our work to existing techniques empirically in a live test-bed and find that our technique outperforms existing techniques.

The rest of the research covers the following sections: Section 2 reviews the related work; Section 3 introduces the detection method; Section 4 explains the experimental setup; Section 5 evaluates the proposed technique; Section 6 discuses the proposed method; and Section 7 concludes the research.

## 2. Related Work

Chen *et al*. [2,3] proposed an approach based on the K-means clustering algorithm to detect MAC address spoofing in WLANs and wireless sensor networks. The authors assume that the RSS samples form a Gaussian. They assume that the RSS samples at a given period at N-sensors form an N-dimensional vector, and the number of clusters is two (*i.e.*, k=2). They then use the Euclidean distance algorithm to compute the distance between the two centroids and eventually detect any MAC address spoofing. In practice, their approach might not work very well, especially when the hacker and legitimate device are close to each other. The centroids of both devices are close to each other, which makes it hard to differentiate the RSS samples that come from the hacker. In addition, their approach struggles with non-Gaussian data distributions. Finally, one device can form two independent clusters, as we explain in the next sections.

Sheng *et al*. [18] proposed to profile legitimate device RSS samples using the Gaussian mixture model (GMM) clustering algorithm. They assume that the RSS samples from a given sender-sensor pair follow a Gaussian and apply a GMM clustering algorithm to detect spoofing. The solution that they propose has some limitations: a non-Gaussian distribution of the RSS samples could occur in real wireless networks because of interference, multi-path fading and absorption effects. As a result, their approach would not perform well, especially in high traffic wireless networks.

Yang *et al*. [27,28] proposed to use the partitioning around medoids approach, also known as the K-medoids clustering algorithm, to detect MAC address spoofing. This algorithm is better than K-means because it is robust against any noise and outliers that the data might contain. However, they have similar assumptions to those in [2,3]. They assume that there are two clusters (*i.e.*, K=2). They also assume that, under normal conditions, the distance between the two medoids should be small because there is only one cluster at a specific location that is the legitimate device. In contrast, under abnormal behavior, the distance between the two medoids should be large, and this suggests the existence of an attacker [27,28]. This approach has a problem that is similar to the K-means-based approach, which is that it is difficult to determine the attacker if he/she is in close proximity to the legitimate device, because the two medoids are close to each other and the RSS samples are mixed together. In addition, one device can have two independent clusters that could degrade the accuracy of their proposed solution.

Sequence number-based approaches [25,26] have been proposed by several researchers exploiting the fact that every data and management frame has a sequence number in the MAC header. The sequence number typically is incremented by one when the sending device sends a management or data frame. The sensor captures the frames from the same MAC address, and if it finds there is a gap between two consecutive frames, it assumes that MAC address spoofing has occurred. These approaches cannot work well when the legitimate station is not sending any frames. In addition, it cannot detect an attacker when it only sends control frames, as control frames do not have sequence numbers.

The authors of [31] proposed a technique to detect MAC address spoofing using the Physical Layer Convergence Protocol (PLCP) header, data rates and modulation types in particular. This technique is used to distinguish between the rogue device and the genuine device. The data rates and modulation types are extracted from the physical layer meta-data (such as RadioTap and Prism) of each captured frame to detect rogue devices. The modulation types and the data rates depend on the rate adoption algorithm. The information that they use to detect spoofing belongs to the physical layer, which makes their approach more robust against spoofing. The only problem with their approach is that it depends on a small number of data rates and modulation types to detect attackers. Thus, it is possible that the attacker uses the same modulation type and the data rate as the legitimate device because they are limited.

Tao *et al*. [24] proposed a layered architecture named wireless security guard (WISE GUARD) to detect MAC address spoofing using three stages. The first stage is OS fingerprinting, which can be applied to the network layer in the protocol stack. The authors extended the synchronization (SYN)-based OS fingerprinting because it is capable of differentiating the attacker from the legitimate device only if the attacker injects data frames into the network. They utilized the capability information, traffic indication map and tag information (which includes the vendor information) to extend it. The second stage employs the data link layer, the sequence number field in particular. They utilized the idea that there could be a sequence number gap between the legitimate device and the attacker consecutive frames. The third stage brings to play the RSS, which belongs to the physical layer; unfortunately, the authors did not explain this stage in much detail.

The authors established some rules to detect the MAC address spoofing. They used a simple and yet effective technique to combine the outputs from the three stages. Every stage outputs either normal or abnormal states of every upcoming frame. They then combined the outputs to evaluate how severe the suspicious frame is; if the analyzer finds the outputs of more than one stage to be abnormal, the alert is triggered. If the OS fingerprinting stage alone is abnormal, the alert is triggered. This indicates that the MAC address of the AP is masqueraded, because the OS fingerprinting that the authors used depends on fields that are vital to the APs, such as capability information. Some drawbacks exist in such approaches: most of the spoofing attacks involve control and management frames, and these frames cannot reveal OS characteristics; therefore, most of the intrusions in WLANs go undetected. OS fingerprinting also assumes that most of the tools that attackers use are based on Linux-based operating systems. This is somehow a valid assumption, but the Windows operating system also provides a capability to change the MAC address of any wireless card in the WLAN. The sequence number techniques have several drawbacks, as explained previously, so combining both SN and OS fingerprinting could miss some intrusions.

## 3. MAC Address Spoofing Detection Method

RSS has been adopted by researchers for localization for several years because of its correlation to the location of a wireless device [32,33,34,35,36,37]. The goal of localization is to focus on RSS samples of a single device. In contrast, in spoofing detection, it is sometimes difficult to distinguish between two devices at different locations that claim to be the owner of a specific wireless device through spatial information alone, especially when they are in close proximity. We exploit the fact that RSS samples at a specific location are similar while the RSS samples at two different locations are distinctive. To distinguish an attacker, we should first develop the characteristics of normal behavior by building a profile of the legitimate device.

### 3.1. Network Architecture

**Figure 1:**
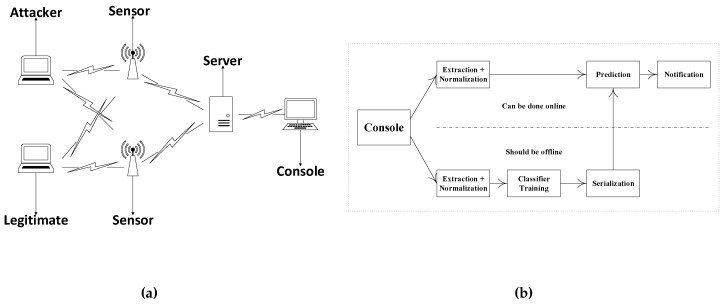
Network architecture and profiling. (**a**) Network architecture; (**b**) profiling and detection.

The network architecture is assumed to be similar to the one that is in Figure 1a, which consists of sensors monitoring the network. Every sensor captures frames from nearby wireless devices. Each sensor sends the important information of the captured packets, as shown in Figure 1a, to the server for global detection. The console receives the packets, normalizes the RSS samples using the timestamps or sequence number, combines the packets and constructs the sample. Each sample contains the information of the same packet from both sensors.

### 3.2. Profiling Based on Random Forests

The proposed framework involves two stages: the offline stage and the online stage. In the offline stage, the legitimate device profile is built. During profiling, we label the legitimate device RSS samples for the training set as zero and all possible other locations as one to construct a profile of the legitimate device. We train the classifier on 50% of the data for each combination (this can be done once per new environment or periodically). We test on 50% of the unseen data to evaluate our predictor. Once we are satisfied with our predictor, we can serialize it, as shown in Figure 1b, to predict new unseen data. After serialization, the training procedure depicted in the lower part of the figure is not necessary for real-time prediction. Thus, in the online stage, any new packet can be fed immediately to the predictor. The predictor then predicts if the packet comes from a legitimate device or not. If it finds that the packet is coming from a suspicious device, an alert is triggered.

Let *x* denote the RSS sample and C denote the class, so that:

$$C=\left\{\begin{array}{cc} 0 & \;\text{if}\;x\;\text{is}\;\text{genuine}\\ 1 & \;\text{if}\;x\;\text{is}\;\text{suspicious} \end{array}\right.$$

Data points are denoted by a vector:

(1) v=(z1,z2,...,zm)

where *z* is an integer representing the signal strength of each frame in the signal space.

Dataset *d* can be represented as:

(2) $$d=\left(x_{1},y_{1}\right),...,\left(x_{n},y_{n}\right)$$

where *d = 20,000* for each combination in Equation (2), $x_{i}$ is the RSS sample and $y_{i}$ is its label.

Additionally, $x_{i}$∈N-dimensional, where N-dimensional is feature vectors having RSS samples captured by each sensor (e.g., the first feature is the RSS samples captured by the first sensor; the second feature is the RSS samples captured by the second sensor, and so on).

We used the Python library [38] in our experiment to train and test our detection method. Algorithm 1 shows the training set using the random forests ensemble method. Random forests uses a specified number of trees (e.g., 100) to perform the whole procedure. Each tree works on a different subset of the dataset randomly to create the ensemble [39].

**Algorithm 1** Training using the random forests algorithm.
1:**for** *t* = 1 to *F* **do**                                   ▷F=100 2:    Uniformly render a bootstrap sample $Z^{*}$ from *d*  3:    Random forests tree $T_{t}$ increases bootstrapped data $Z^{*}$ in size by performing the following steps:
  At each node, choose *r* features randomly    Choose the best possible feature        ▷$x_{i}\in N-\mathrm{dimensional}$ as stated previously   Split into two child nodes using the best split-point                 ▷r⩽N 4:    **Output:** Trees ensemble ${\left\{T_{t}\right\}}_{1}^{F}$ 5:**end** **for**


To detect MAC address spoofing, we used the prediction ability of random forests after serialization to predict unseen new samples, as indicated in Algorithm 2. The new sample is classified as normal or abnormal, if the predictor finds it to be different from the profiled samples.

**Algorithm 2** Detection algorithm.
1:**for** profiled MAC address frames **do** 2:    Predict every sample using the following equation      ▷ To predict new data point $\hat{x}$
(3)$$c_{R}^{F}=M_{v}{c_{t}\left(\hat{x}\right)}_{1}^{F}$$       ▷$c_{t}$ is the prediction class of the random forests      ▷$M_{v}$ is the majority vote 3:    **If** the sample is different from the legitimate device samples 4:    **Output:** A rogue device has been detected 5:**end** **for**


## 4. Experimental Section

**Figure 2:**
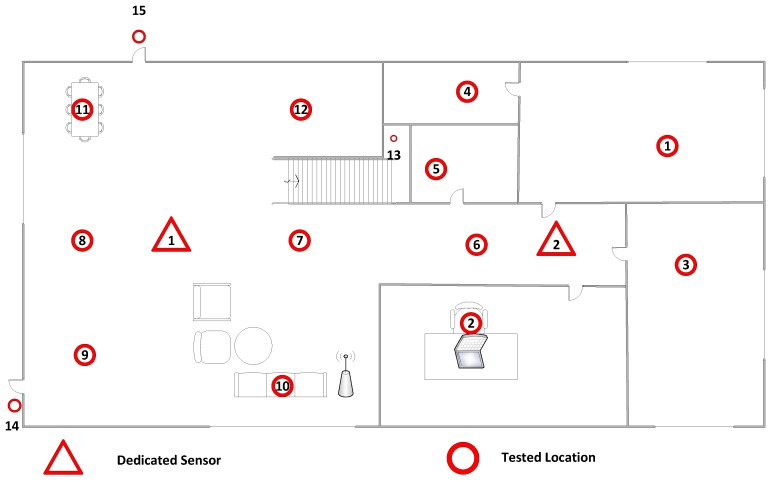
Test-bed.

We covered an area of 102 m${}^{2}$ using 15 locations marked by the red dots in Figure 2 to evaluate our proposed method. The distance between any two neighbors is about four meters (from 3–5 m). We tried to simulate the attacker to be at every possible place throughout our test-bed. We placed two sensors, indicated by the triangles, to cover as much ground as possible of the network diameter. We also used some active probing techniques to force the device to respond to specific frames in order to speed up the process of profiling. Each sensor captures enough packets for legitimate device profiling. The total number of combinations is 105; we chose one location to be the location of the legitimate device (e.g., Location 1), picked another location for the suspicious device (e.g., Location 2) and ran the test for all other locations (e.g., Locations 3–15) as the attacker against the legitimate device (*i.e.*, Location 1), as well. We tested all possible combinations.

### 4.1. Hyperparameter Optimization

**Figure 3:**
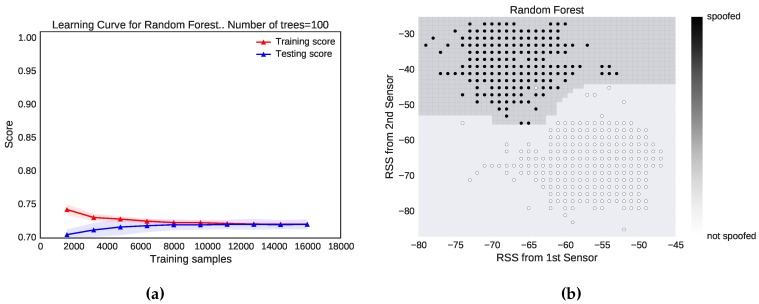
Optimization and data separation. (**a**) Learning curve of random forests with 100 trees for Locations 6 *vs.* 7; (**b**) performance of random forests when the attacker and legitimate device are 10 m apart.

To avoid high variance and determine whether the dataset is sufficient to train a random forests classifier of 100 trees, we used the learning curve of one of the noisiest datasets, that of Locations 6 and 7, where the distance between the two locations is less than 4 m, shown in Figure 3a. We started with about 3000 samples and determined that we could improve the accuracy and reduce the variance. At about 15,000 samples, the variance was eliminated and stabilized, indicating that a dataset of 20,000 observations is more than enough. Figure 3b shows how random forests of 100 trees separate the data-points when the attacker is 10 m away from a genuine user. The random forests ensemble method performed very well in the presence of outliers and can separate data of any shape.

### 4.2. Signal Strength Attenuation

**Figure 4:**
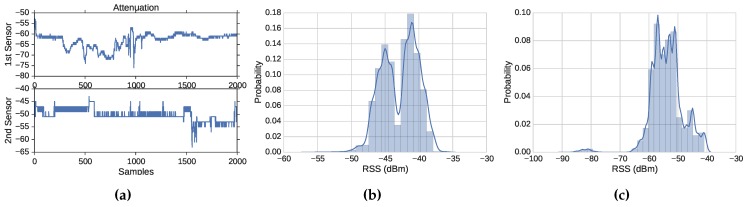
Data distribution and attenuation. (**a**) Attenuation; (**b**) Location 8: Sensor 1 data distribution; (**c**) Location 8: Sensor 2 data distribution.

Figure 4a illustrates the signal attenuation that signal strength might face in wireless networks. We picked two of the sampled locations to represent this phenomenon and measure 2000 consecutive packets at each location. One sampled location is close to the first sensor, and the other one is close to the other sensor. The two subplots show an attenuation of about a 3.4 dB standard deviation (a maximum of −52 dB and a minimum of −76 dB) for the first sampled location and a 2.4 dB standard deviation for the second sampled location (a maximum of −43 dB and a minimum of −63 dB). It is not rare to see some signal attenuation in our experiments. This phenomenon exists because of several factors, such as multi-path fading and obstacles that could make the signal oscillate, especially when there is a significant distance between the sender and receiving device.

The distribution of the data from Location 8 at the two sensors is shown in Figure 4b,c. Some researchers state that the distribution of the transmitter and sensor pair is Gaussian [2,3], while other researchers report that the distribution is not Gaussian [29,30] or that it is not rare to see non-Gaussian distributions of RSS samples [18]. We found that non-Gaussian distributions are not rare and have different distribution shapes and peaks. The distribution of 10,000 RSS samples is shown in the figure. Figure 4b shows a distribution of data that form two Gaussians with one peak that is slightly greater than the other one (*i.e.*, one device has formed two separate clusters), while Figure 4c shows a distribution of data with one Gaussian and some sporadic data points that are far away from the Gaussian. This suggests that using clustering algorithm-based approaches [2,3,18,27,28] can generate many false alerts or cause the intrusion detection system to allow large margins that permit attackers to harm the network.

## 5. Results and Evaluation

To evaluate our proposed solution and compare it to previous work [2,3,18,27,28], we implemented the four possible GMM kernels, because the kernel that [18] used was not indicated in their article. We considered only the best performing kernel (*i.e.*, GMM-full) for comparison. We first calculated the accuracy of the previously-proposed solutions [2,3,18,27,28] along with our proposed method. The clustering algorithm-based approaches [2,3,18,27,28] did not work well, as shown in Table 1a, especially when the two locations were close to each other because of the reasons mentioned earlier (see Section 4.2).

Our proposed method achieved the best accuracy of 94.83. We tested all of the detection methods where the distances between the two locations were less than 4 m, as shown in Table 1b, between 4 and 8 m, as shown in Table 1c, and between 8 and 13 m, as shown in Table 1d. When the locations are close to each other, the clustering algorithm-based approaches [2,3,18,27,28] did not perform well, with a minimum of 47.18% accuracy for Sheng *et al*.’s approach [18], as shown in Table 1b. All of the techniques did slightly better when the locations were a little further apart, as shown in Table 1c. However, all of these methods did very well; our method’s performance remains high when the distance between the two locations increases, as shown in Table 1d.

### Performance Measures

**Figure 5:**
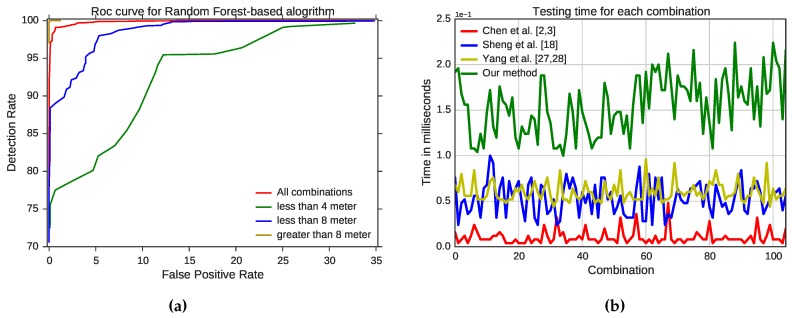
ROC curve of the proposed method and testing time of all of the methods. (**a**) ROC curve for the proposed method; (**b**) testing time for 10,000 samples for the tested methods.

To evaluate our detection method more rigorously, we used the receiver operating characteristic (ROC) curve, shown in Figure 5a, which plots the detection rate, that is the true positive rate or sensitivity against the (1 - specificity) or false positive rate (FPR). We evaluated our detection method to measure the tradeoff between correct detection and FPR for different distances between the attacker and legitimate device. At 3% FPR, the correct detection rate is about 99% for all combinations in our test-bed. At 12% FPR, the detection rate is 99% when the distance between the attacker and legitimate device is between 4 and 8 m. At 25% FPR, the detection rate is 90% when the distance between the attacker and the legitimate device is less than 4 m and 100% when the distance is between 8 and 13 m. We also measured the prediction time to see if it is possible to predict the captured frames in real time. Table 2 shows the average testing time, standard deviation, minimum and maximum values for 10,000 samples of all of the tested locations. The clustering algorithm-based methods, of Chen *et al*. [2,3], Sheng *et al*. [18] and Yang *et al*. [27,28], are faster than our method. Chen *et al*.’s [2,3] approach is the fastest with times as high as 48 ms. Figure 5b illustrates the overall performance of our detection method and the existing methods with regard to testing time. Our detection method has a good performance in terms of testing time, with an average of only 155 ms.

## 6. Discussion

RSS measurements can be utilized to differentiate wireless devices based on location. Some factors play a vital role in measuring the RSS, such as multi-path fading, absorption effects, transmission power and the distance between the transmitter and the receiver. Our experiment shows multiple situations where the data forms different shapes and peaks. This is probably because WLAN devices interfere with one another. In addition, microwave ovens and Bluetooth might cause more collision and interference in the frequency band. Thus, our proposed method is very effective because (unlike the previous solutions [2,3,18,27,28] that could deal with the data if it were only Gaussian distributed) our method could pick the data of any shape. The overall accuracy of our proposed method is 94.83% of all combinations, which outperforms the previous solutions: the overall accuracy of Chen *et al*.’s [2,3] solution is 88.95; the accuracy of Sheng *et al*.’s [18] solution is 87.59%; and the accuracy of Yang *et al*.’s [27,28] solution is 91.17%.

We tested the proposed method where the distances between the genuine device and the attacker are less than 4 m, from 4–8 m and from 4–8 m. The longest distance between any two locations in our test-bed is about 13 m. Although we did not test any two locations where the distance is more than 13 m, we believe that the accuracy would be perfect as the distance between the attacker and the legitimate device increases to more than 13 m. We also did not test different types of antennas, such as directional or beam antennas, because this research assumes that the attacker uses an omnidirectional antenna, so more sophisticated attacks might remain undetected.

**Figure 6:**
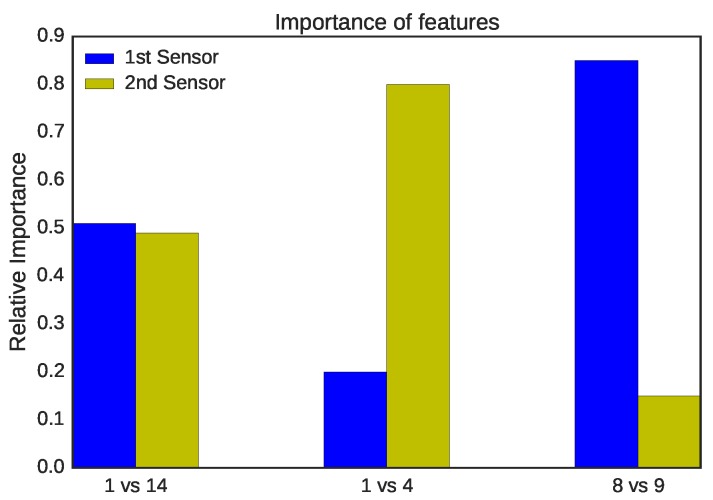
Feature importance of three tested combinations.

The sensors placement is significant to determine the difference between the profiled legitimate device samples and the masquerader frames. Figure 6 shows how important the features after training are at determining the two locations for three different combinations (note that understanding feature importance is a capability that is provided by almost all of the ensemble methods). The first feature comprises the RSS samples captured by the first sensor, and the second feature consists of the RSS samples captured by the second sensor. The figure shows which sensor determines most of the samples of Locations 1 and 14. It appears that the two sensors are close: about 51% are determined by the first sensor and 49% by the second sensor. In this case, the distance between the attacker and the legitimate device is about 12 m. The legitimate device (*i.e.*, Location 14) is 3 m from the first sensor. The hacker (*i.e.*, Location 1) is about 9 m away from the first sensor and about 3 m away from the second sensor.

Locations 1 and 4 are both close to the second sensor, so the second sensor determines most of the samples (about 80%), as shown in the figure. Location 4 is about 5 m from Sensor 2 and is about 11 m from Sensor 1. In addition, the distance from the attacker to the legitimate device is about 4 m. Locations 8 and 9 are close the first sensor; thus, the first sensor determines which samples belong to which class for the majority of samples (about 85%), as shown in the figure. Location 8 is about 2 m away from the first sensor and about 10 m away from the second sensor. Location 9 is about 4 m away from the first sensor and 11 m away from the first sensor. The two locations are about 4 m away from each other.

## 7. Conclusions

In this article, we proposed a technique based on the random forests ensemble method, which characterizes the shape of a dataset to detect MAC address spoofing, instead of assuming that the data are Gaussian distributed. All previous methods based on clustering algorithms assume that there are two clusters, which is not a valid assumption, because one device, such as an AP, can form two clusters. Based on our extensive experiments and evaluations, we determined that our proposed method performs very well in terms of accuracy and prediction time. We proposed a technique to detect MAC address spoofing based on random forests, as it outperforms all of the clustering algorithm-based approaches that were proposed previously, in terms of accuracy. Furthermore, it has a good prediction time. In our future work, we will consider an outlier or novelty detection method to detect MAC address spoofing. Outlier/novelty detection methods only require training using a legitimate device without covering the whole network range. We plan to use an approach that is based on a one-class SVM to build a profile for legitimate devices.

## Figures and Tables

**Table 1:** Detection accuracy by distance between locations.

	Chen *et al*. [2,3]	Sheng *et al*. [18]	Yang *et al*. [27,28]	Our Method
Mean	88.9492	87.5902	91.1658	94.8296
std	14.0435	15.2362	11.0422	7.1087
Min	53.38	28.61	53.21	71.35
50%	96.08	94.95	96.47	98.81
75%	98.75	99.53	98.76	99.92
Max	100	100	100	100
(**a**) All location combinations (105 combinations).				
	**Chen *et al*. [2,3]**	**Sheng *et al*. [18]**	**Yang *et al*. [27,28]**	**Our Method**
Mean	76.5895	76.3920	80.3875	88.3800
std	15.4416	15.2714	13.5181	8.2278
Min	53.41	47.18	53.21	75.88
50%	77.520	70.375	81.345	89.640
75%	89.3675	90.6650	90.9975	94.5825
Max	98.56	98.25	98.56	99.77
(**b**) Locations <4 m apart (20 combinations).				
	**Chen *et al*. [2,3]**	**Sheng *et al*. [18]**	**Yang *et al*. [27,28]**	**Our Method**
Mean	85.7275	82.5584	89.1618	93.1614
std	14.2020	16.0099	10.0814	6.8342
Min	53.38	28.61	64.56	71.35
50%	91.360	84.740	92.600	95.610
75%	96.2825	96.5850	96.9475	98.6025
Max	99.72	99.91	99.72	99.95
(**c**) Locations >4 m and <8 m apart (44 combinations).				
	**Chen *et al*. [2,3]**	**Sheng *et al*. [18]**	**Yang *et al*. [27,28]**	**Our Method**
Mean	98.4359	98.4527	98.5741	99.7661
std	1.6246	2.3989	1.4843	0.42908
Min	94.31	92.04	94.97	98.22
50%	99.09	99.72	99.09	99.95
75%	99.76	99.94	99.76	99.98
Max	100	100	100	100
(**d**) Locations is >8 m and <13 m apart (41 combinations).				

**Table 2:** Testing time for all location combinations.

	Chen *et al*. [2,3]	Sheng *et al*. [18]	Yang *et al*. [27,28]	Our Method
Mean	0.010400	0.053219	0.060190	0.154705
std	0.007718	0.017691	0.010918	0.031848
Min	0.004	0.024	0.044	0.100
Max	0.048	0.100	0.096	0.224
